# Patterns in clinical chemistry requests

**DOI:** 10.1155/S146392468900012X

**Published:** 1989

**Authors:** Jan B. Hemel, Frans R. Hindriks, Hilko van der Voet, Leo R. Rijnveld

**Affiliations:** 1 Central Laboratory for Clinical Chemistry University Hospital Groningen PO Box 30001 Groningen NL 9700 RB The Netherlands; 2 Research Group Chemometrics Pharmaceutical Laboratories State University of Groningen A. Deusinglaan 2 Groningen NL 9713 AW The Netherlands; 3 Department of Information and Automation University Hospital Groningen PO Box 30001 Groningen NL 9700 RB The Netherlands; 4 University of Groningen Computing Centre PO Box 800 Groningen NL 9700 AV The Netherlands; 5 Agricultural Mathematics Group PO Box 100 Wageningen NL-6700 AC The Netherlands

## Abstract

For each patient sample that is presented to the clinical chemistry laboratory a combination of various tests can be requested. This combination or profile will depend on the condition of the patient, and hence also on the requesting hospital department. Several techniques were applied to detect and describe patterns in tests requested by the cardiology, hepatology and nephrology sections of the out-patient's Department for Internal Medicine. Comparison of the frequencies of ordering the tests showed significant differences between these sections. Cluster analysis and multidimensional scaling were used to show similarities and differences in the test profiles that were used by the sections. These techniques are useful for generating hypotheses, but the statistical significance of the clustering found is difficult to assess.


Journal of Automatic Chemistry Vol. 11, No. 2 (March–April 1989), p. 55–63

### Patterns in clinical chemistry requests

**Jan B. Hemel,† Frans R. Hindriks**
*Central Laboratory for Clinical Chemistry, University Hospital Groningen, PO Box 30001, NL 9700 RB Groningen, The Netherlands*

**Hilko van der Voet‡**
*Research Group Chemometrics, Pharmaceutical Laboratories, State University of Groningen, A. Deusinglaan 2, NL 9713 AW Groningen, The Netherlands*

**and Leo R. Rijnveld**
*Department of Information and Automation, University Hospital Groningen, PO Box 30001, NL 9700 RB Groningen, The Netherlands*

*For each patient sample that is presented to the clinical chemistry laboratory a combination of various tests can be requested. This combination or profile will depend on the condition of the patient, and hence also on the requesting hospital department. Several techniques were applied to detect and describe patterns in tests requested by the cardiology, hepatology and nephrology sections of the out-patient's Department for Internal Medicine. Comparison of the frequencies of ordering the tests showed significant differences between these sections. Cluster analysis and multi-dimensional scaling were used to show similarities and differences in the test profiles that were used by the sections. These techniques are useful for generating hypotheses, but the statistical significance of the clustering found is difficult to assess.*

### Introduction

In recent years the increase in spending on public health has attracted much attention. The abundance of facilities for diagnosis and monitoring may have improved medical care, but has also tended to increase the costs of medical decisions. Great efforts are put into controlling these costs, and of course careful analyses of the structure of the expenditure involved with medical decisions is a pre-requisite for controlling them. This paper discusses some tools for making these analyses and then applies them to studies on chemistry blood test requesting.

Clinical chemistry tests are indispensable for assessing diagnoses and for monitoring the course of a disease. For every patient a selection must be made from a wealth of available tests. At first sight it may seem appropriate to select the clinical chemistry tests separately for each patient. However, it is clear from practice that systematizing, for example in the form of test request schemes, enhances efficiency, both for the physician and the laboratory. Experience with the interpretation of a well-established set of tests will help the physician, and dedicating analytical apparatus to frequently requested test profiles speeds up the laboratory processing, and may decrease laboratory costs. Today, a substantial proportion of analytical apparatus used in the clinical chemistry laboratory (chromatographic equipment and multichannel analysers for example) yields multiple results, even if only few of these results are requested. The configuration of this apparatus can often be modified in order to minimize production of unrequested test results. The capacity of a clinical chemical laboratory should be carefully tuned to diagnostic needs of the medical staff. Because of the progress in both medicine and analytical chemistry, updating of laboratory equipment is necessary from time to time. Test profiles that are offered to the physician as standard may be replaced. From a laboratory management point of view, it is an interesting task to adapt the equipment to the needs, and vice versa the needs to the equipment, maintaining high standards and reducing laboratory costs.

This paper describes the test profiles used by different groups of physicians at Groningen's University Hospital, and shows the differences between these profiles. The in-patient department was compared to the out-patient department, and the cardiology, hepatology and nephrology sections of the out-patient Department for Internal Medicine were compared according to a pairwise scheme.

Similar problems of finding groups of related objects occur also in other fields of clinical chemistry. The degree to which diseases (or diagnoses) occur simultaneously could be approached in a similar way, as well as the composition of Diagnosis-Related Groups (DRGs), which promise to become a major tool for controlling health-care costs.

After describing the data in the next section, two types of statistical analysis are discussed: the classical type to test hypotheses, and the descriptive one where displays are made by applying techniques from non-supervized pattern recognition, namely cluster analysis and multi-dimensional scaling. In the last sections, the results are presented and discussed.

### Data

The study was based on the 30 tests that are performed most frequently in our clinical chemistry laboratory, (see table 1). The work covered all blood sample test requests submitted to our laboratory within a period of 100 days, involving more than 400 000 tests. Each request can be regarded as a sequence of 30 0s and 1s, the 1s indicating that the corresponding test is to be performed, and the 0s denoting the tests to be omitted.

### Methods

Differences in the test profiles were studied initially by comparison of frequencies of requesting each test.

† Present address: University of Groningen, Computing Centre, PO Box 800, NL 9700 AV Groningen, The Netherlands.

‡ Present address: Agricultural Mathematics Group, PO Box 100, NL-6700 AC Wageningen, The Netherlands.

0142–0453/89 $3.00 © 1989 Taylor & Francis Ltd.

*Table 1. Abbreviations used in this paper.*

| | |
|---|---|
| ALL | Test requests from all hospital departments |
| CAR | Test requests from the out-patient cardiology department |
| HEP | Test requests from the out-patient hepatology department |
| IN | Test requests from all in-patient departments |
| NEP | Test requests from the out-patient nephrology department |
| OUT | Test coming from all-patient departments |
| ACP | Acid phosphatase |
| AST | Aspartate amino transferase |
| ALT | Alanine amino transferase |
| ALB | Albumen |
| AMY | Amylase |
| AP | Alkaline phosphatase |
| CA | Calcium |
| CHE | Cholinesterase |
| CHO | Cholesterol |
| CMB | Creatine kinase MB-fraction |
| CL | Chloride |
| CK | Creatine kinase |
| CR | Creatinine |
| DBI | Direct bilirubin |
| FE | Iron |
| GGT | γ-glutamyl transferase |
| GLU | Glucose |
| K | Potassium |
| LD | Lactate dehydrogenase |
| MG | Magnesium |
| NA | Sodium |
| P | Inorganic phosphate |
| PAP | Prostate acid phosphatase |
| PEP | Protein electrophoresis |
| TBI | Total bilirubin |
| TGL | Triglycerides |
| TIB | Total iron binding capacity |
| TP | Total protein |
| UA | Uric acid |
| UR | Urea. |

The following pairwise comparisons were made: IN versus OUT, CAR versus HEP, CAR versus NEP, and HEP versus NEP (for abbreviations see table 1). The test-statistic $T_{ij}$ used to compare frequencies of each single test was based on a normal approximation for binomially distributed variables:

$$T_{ij} = \frac{(f_{i1}/n_1 - f_{i2}/n_2)}{\sqrt{(p_0(1-p_0)(1/n_1 + 1/n_2))}}$$

where $f_{ij}$ = the frequency in section $j$ of requesting test $i$, $n_j$ = the total number of tests requested in section $j$, $j$ (= 1 or 2) = coding for either one of IN, OUT, CAR, HEP and NEP, and $p_0 = (f_{i1} + f_{i2})/(n_1 + n_2)$.

Under the null-hypothesis that no differences exist in the population frequencies, $T_{ij}$ is approximately standard-normally distributed, and the significance of the difference can thus be easily assessed. The test is an asymptotic version of Fisher's exact test for the comparison of two probabilities.

These studies do not answer the question about which tests are requested simultaneously, in other words what test profiles are used in the various hospital departments. To shed some light on these profiles, a cluster analysis was carried out.

Cluster analysis relies heavily on the definition of the distance measure that is used. Conclusions only hold as far as this measure is acceptable. In choosing the measure's definition one should therefore consider the aims of the clustering. The main aim of clustering in this study is detecting groups of tests that behave like an entity in test requests: the entire group is either requested or completely omitted from the request. In the following a distance measure is described which detects this behaviour. Clusters obtained using different clustering criteria and distance measures are generally not comparable.

Two tests requested for the same sample, as well as tests that are simultaneously not requested, are candidates for the same cluster, because they seem to behave like an entity. Their distance to each other is defined as zero. On the other hand, two tests of which only one is requested, are obviously bad candidates for the same cluster; their distance is arbitrarily set to unity. From $n$ test requests (with choice from $p$ tests) a $p \times p$ Euclidean distance matrix $\mathbf{D}$ was computed as $\mathbf{D} = (\Sigma_{i=1}^{n} \mathbf{D}_i)^{1/2}$, where $\mathbf{D}_i$ is a $p \times p$ distance matrix for request $i$ with elements $d_i(j,k)$ equal to 0, if tests $j$ and $k$ were both ordered or both not ordered, and equal to 1, if only one of them was ordered. The matrix $\mathbf{D}$ is a matrix of city-block or Hamming distances between the tests [1]. In this special case these distances are equal to the squared Euclidean distance. The distances in this matrix can be interpreted as follows. If test $a$ and test $b$ were offered exclusively as a package, the number of tests that was performed unnecessarily in the period under study is given by the distance matrix element $\mathbf{D}_{a,b}$.

Starting with a distance matrix, several techniques can be applied to identify clusters [2–6]. One of the more popular ones is Ward's hierarchical clustering [2 and 7]. This technique starts by considering each test as a separate cluster. Subsequently, in each step those two clusters whose agglomeration causes a minimum loss of structure are combined. For an exact definition of the criterion used the reader is referred to the literature [2 and 7].

Agglomeration continues until only one cluster results. A dendrogram showing all steps of the clustering process can be drawn. This is a graph with an inverted-tree shape, each branch downward splitting up into two sub-branches. The 'natural' number of clusters follows from the loss of structure in each agglomeration step, represented in the dendrogram as the length of the linking lines. Large 'steps' correspond to large differences between the sub-clusters that are linked. The hierarchy of this technique implies that objects cannot be relocated to another cluster during the clustering process. Ward's method was used to detect clusters and differences between them in the test profiles in use in the hospital departments under study.

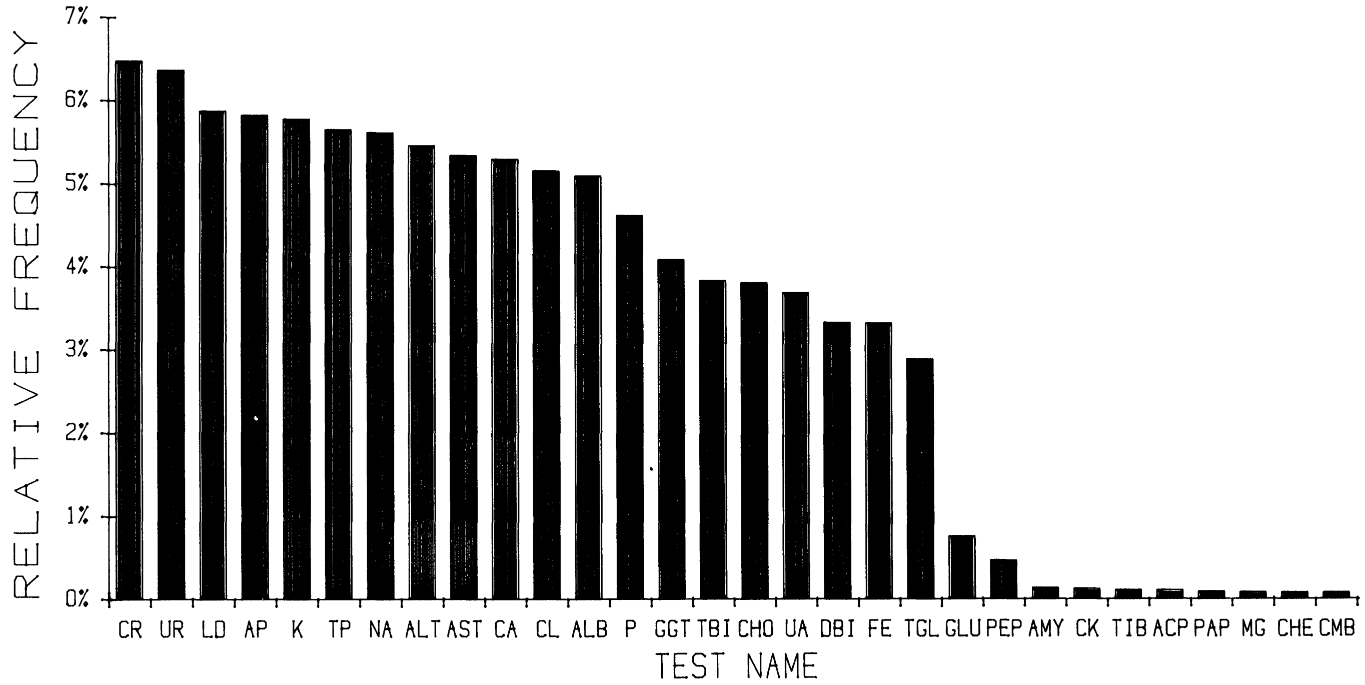

*Figure 1. Relative frequencies of clinical chemistry test requests in the entire hospital. For abbreviations see table 1.*

Also, display techniques like the Karhunen-Loève plot (based on principal components analysis [PCA]), and multidimensional scaling (MDS) [8], are suited to suggesting frequently occurring combinations of tests. Techniques that are based on principal components require the original matrix consisting of all test requests to be available. In our case this matrix would be too large for processing, therefore from this matrix only the absolute frequencies of performing each test and the distance matrix were calculated and retained. Multidimensional scaling can be based on a distance matrix without the original data matrix being known. The applied MDS technique creates a two-dimensional map of the laboratory tests on the basis of their mutual distances. A higher-dimensional space may be needed to allow for a more accurate representation of the distances between the 30 tests as collected in the distance matrix. The tests can be regarded as thirty points, usually occupying a 29-dimensional subspace of the $n$-dimensional space spanned by the $n$ observed requests. Forcing this multidimensional space into two-space (mapping it) usually involves distortion of the inter-point distances. By multidimensional scaling this distortion is minimized to obtain a map reflecting the mutual test distances best. Several criteria for 'goodness of fit' exist. The maps yielded by the Kruskal minimal stress criterion [8 and 9] were created for each department's distance matrix and visually interpreted.

### Materials

The Hospital Information System used for data retrieval was BAZIS [10].

Cluster analysis was performed using the CLUSTAN computer program package [3], running on a Control Data Cyber 170/760 computer at Groningen State University.

For multidimensional scaling the program SYSTAT 3·0 [11] was employed, running on an IBM-AT microcomputer.

### Results and discussion

Figure 1 shows the frequencies of ordering each test, in the hospital as a whole. The frequencies are expressed as a percentage of the total number of tests ordered. The abbreviations used for the tests are given in table 1. A selection of 20 tests (from CR to TGL) makes up the bulk of the work-load. In fact these 20 tests are offered as a general-purpose screening package, performed on a single continuous-flow analyser. The large difference in frequency between these 20 tests and all other tests poses some problems. Small distances between relatively frequently ordered tests are largely determined by their simultaneous presence, while simultaneous *absence* is the main cause that rare tests are close. This leads to an interpretation problem with distances between frequently and rarely ordered tests: do large distances originate from the physician needing only one of the two tests, because both tests offer the same information; or is one of the tests serving such specific purposes that it is seldom needed? As efficiency is usually less of a problem with uncommonly ordered tests, only the 20 most common tests were included in the rest of this study to avoid this ambiguity.

There is a real difference in test ordering between in- and out-patient departments – this is clearly seen in figure 2. In this plot the difference $\mathrm{Diff}_{i,\mathrm{IN},\mathrm{OUT}}$ between the

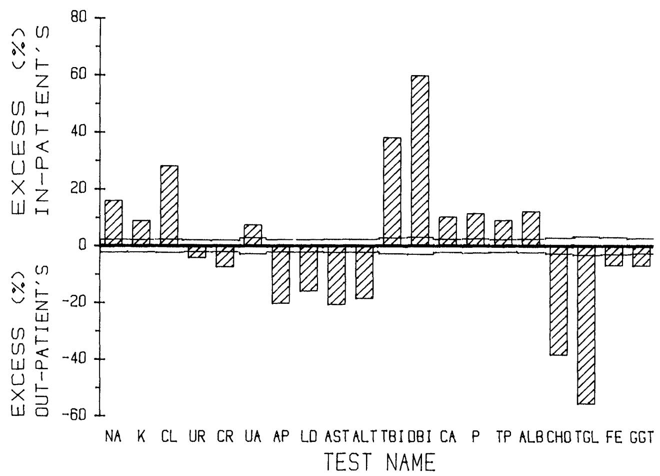

*Figure 2. Differences in frequency of test requests between in-patient and out-patient departments – level $p = 0{\cdot}05$.*

relative frequencies of test ordering of these sections is shown, expressed as a percentage of the mean relative frequency. In formula notation

$$\mathrm{Diff}_{i,\,\mathrm{IN,OUT}} = \frac{(f_{i,\,\mathrm{IN}}/n_{\mathrm{IN}} - f_{i,\,\mathrm{OUT}}/n_{\mathrm{OUT}})}{(f_{i,\,\mathrm{IN}} + f_{i,\,\mathrm{OUT}})/(n_{\mathrm{IN}} + n_{\mathrm{OUT}})}\,100\%$$

where $f_{i,j}$ ($j$ = IN, OUT) = the frequency in section $j$ of requesting test $i$ and $n_j$ = the total number of tests requested in section $j$.

The lines that are almost horizontal indicate the critical levels ($p = 0{\cdot}05$). The bars pointing up indicate that the test is requested more for admitted patients, while down-pointing bars show more out-patient requests. Particularly prominent is that the bilirubins are most often requested by the clinic, whilst most CHO and TGL requests come from the the out-patient department. All other differences are also seen to be significant.

Pairwise plots of the differences between the out-patient departments for cardiology, nephrology and hepatology are shown in figure 3. These plots are analogous to figure 2. Again, many significances are seen, some of which will be briefly discussed.

Hepatologists are more interested in bilirubin and GGT than cardiologists (figure 3[*a*]); these tests find their main use in indicating diseases involving the biliary tract. Impaired protein synthesis may indicate liver cell damage, therefore hepatologists ask for the ALB and TP tests relatively often. The frequent requests of the lipid profile (CHO and TGL) by cardiologists can be explained by the role of lipids in atherosclerosis. The cardiological interest in kidney function and the importance of electrolytes for the cardiac function explains the upward pointing bars of kidney function and electrolyte tests (UR, CR, NA, K and CL).

It is interesting to note (figure 3[*b*]) that no great difference in interest exists between cardiologists and nephrologists in kidney function. Enzyme tests (LD, ALT, AST) as indicators for cardiac muscle damage are often ordered by cardiologists, while nephrologists request tests for proteins (TP and ALB; hydration), calcium and phosphate (renal calculi) relatively often.

The hepatologists and nephrologists appear to adhere strongly to their specialisms when ordering blood tests (figure 3[*c*]).

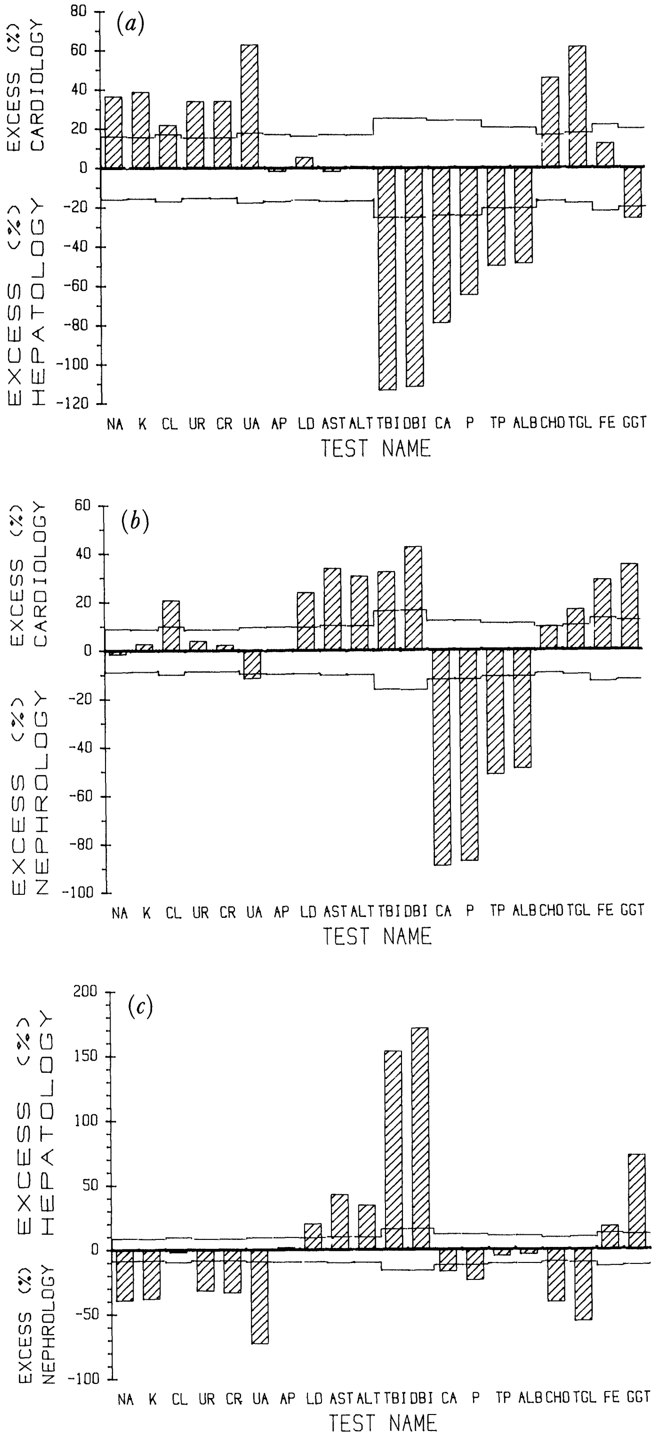

*Figure 3. (a) Differences in frequency of test requests between the cardiology and hepatology out-patient departments. (b) Differences in frequency of test requests between the cardiology and nephrology out-patient departments. (c) Differences in frequency of test results between the hepatology and nephrology out-patient departments. $p = 0{\cdot}05$ in all instances.*

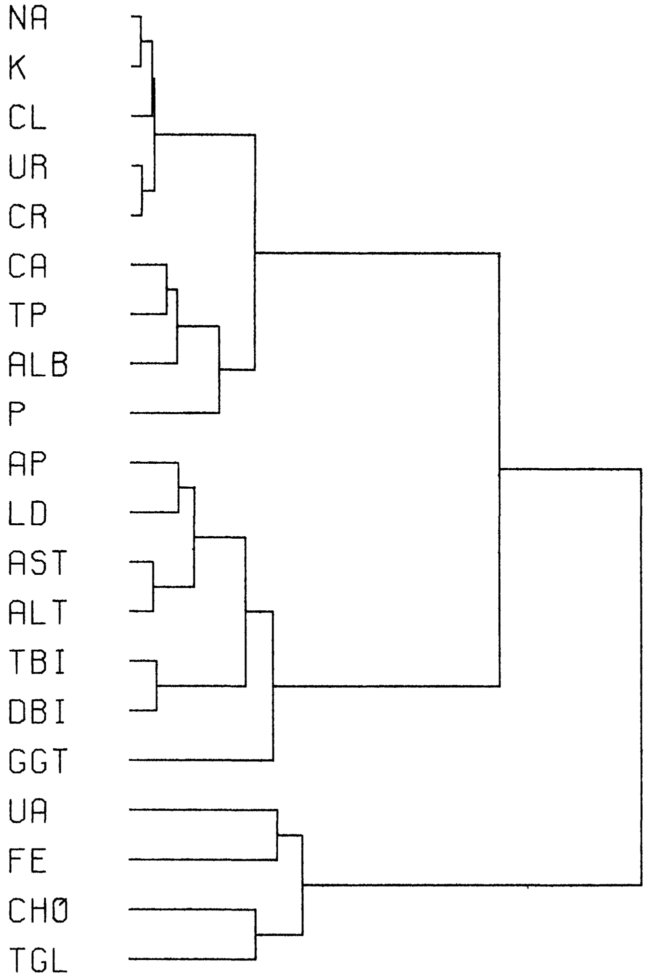

*Figure 4(a). Clusters in test requests of in-patient departments.*

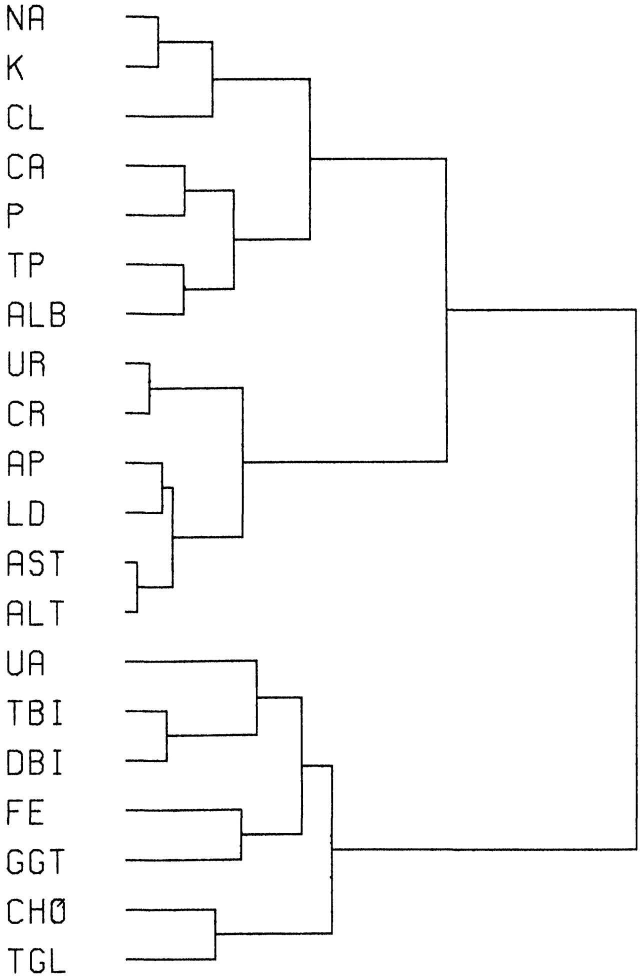

*Figure 5(a). Clusters in test requests of out-patient departments.*

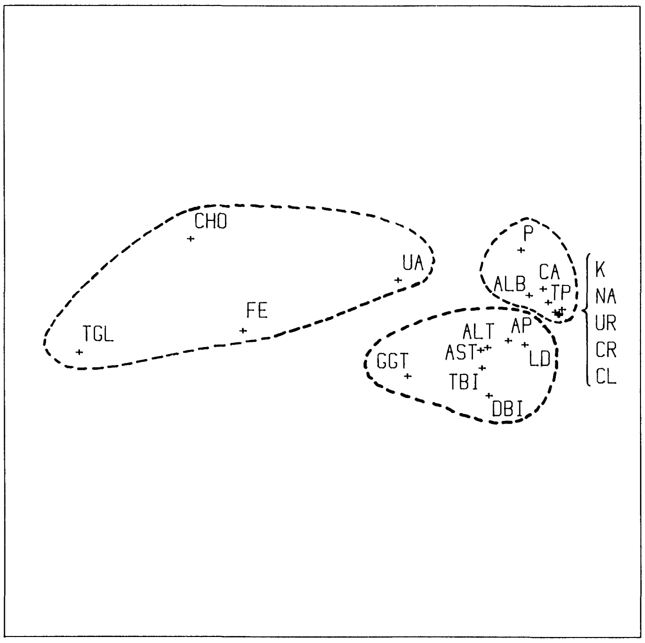

*Figure 4(b). Multidimensional scaling of test requests of in-patient departments.*

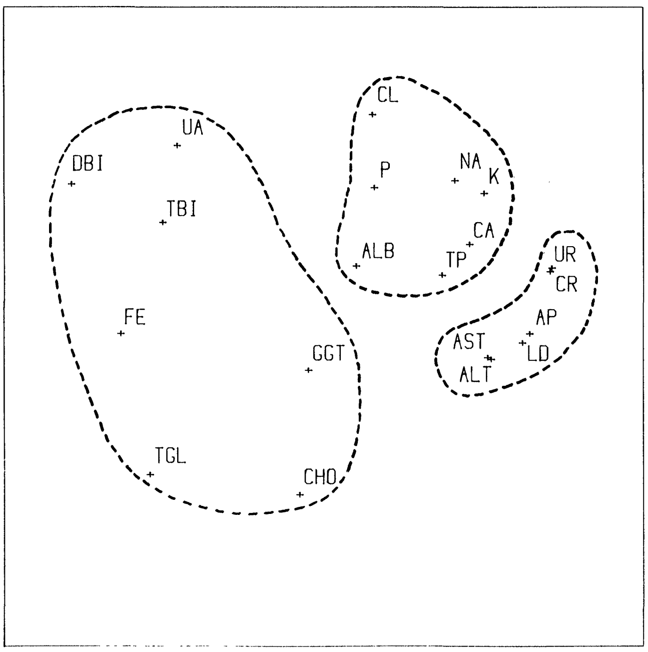

*Figure 5(b). Multidimensional scaling of test requests of out-patient departments.*

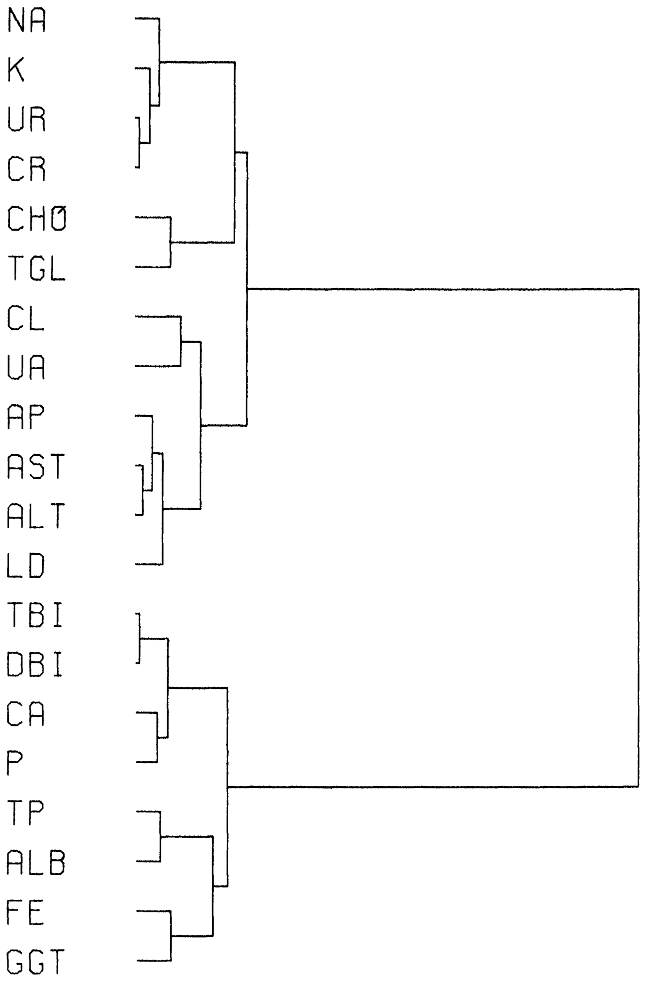

*Figure 6(a). Clusters in test requests of the cardiology out-patient department.*

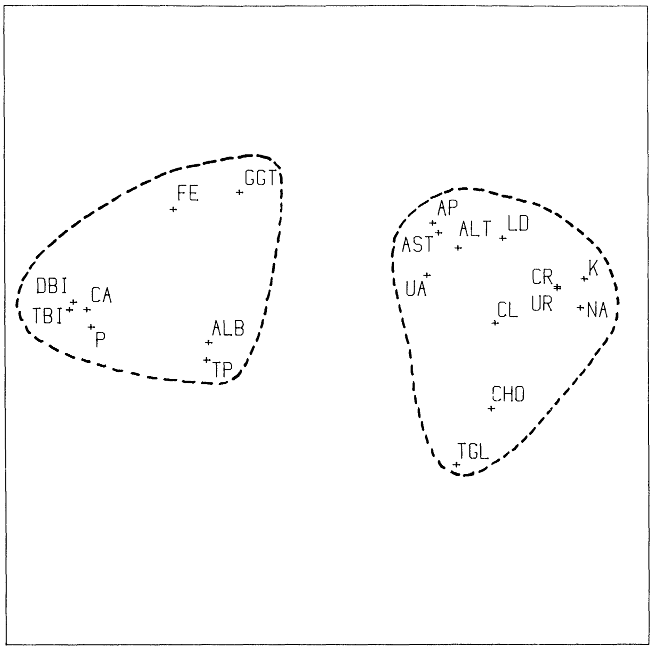

*Figure 6(b). Multidimensional scaling of test requests of cardiology out-patient department.*

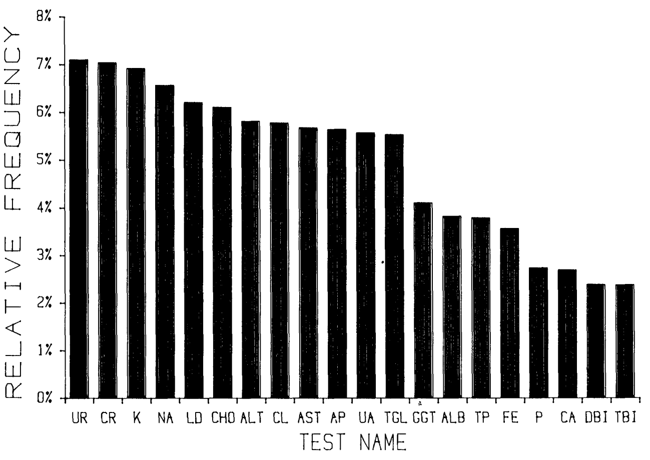

*Figure 6(c). Frequency histogram of test requests of the cardiology out-patient department.*

Clustering of the tests is shown in figures 4 to 8, together with the maps created by multidimensional scaling (MDS). In the MDS maps the main clustering found by Ward's method is depicted by dashed lines. Some tests can be called twins, being included or omitted together in the test request most of the time in all hospital departments. These include urea and creatinine, sodium and potassium, AST and ALT, calcium and phosphate, total and direct bilirubin, albumen and total protein, cholesterol and triglycerides. The differences between the clusters built from these elements in the various departments is discussed in the following.

Differences in test clusters between the out-patient department and the wards can be seen in figures 4 and 5. Chemical tests are ordered in the out-patient's much more on an individual basis, resulting in less clear clustering, than in the in-patient's. This may be a result from greater influence of test request schemes in the wards, and possibly reflects also a broader spectrum of medical decision problems in the out-patient's. Tests for the in-patients are ordered in three main clusters. In these several meaningful sub-clusters are discovered, like a set to detect kidney disease (NA, K, CL, UR and CR), a set informative for liver defects (AP, LD, AST, ALT, TBI, DBI and GGT), and a loosely clustering remaining group (UA, FE, CHO and TGL). The vague main clusters from the out-patient's are very different in composition.

In figure 6 structures in the test series ordered by cardiologists for out-patients are shown. The two main clusters correspond to the tests occurring with a frequency of more and less than 5%, respectively. Within the cluster of the more frequent tests a kidney function sub-cluster, a cardiac muscle (and liver) damage cluster, and a fat metabolism cluster can be detected, all expected to be of interest to a cardiologist. The clustering can also be read from the frequency diagram (figure 6[*c*]). The steps in the slope of this diagram correspond to a considerable extent to the clusters found by Ward's method. In this way evidence is acquired suggesting that the cardiologists proceed according to schemes in requesting laboratory tests.

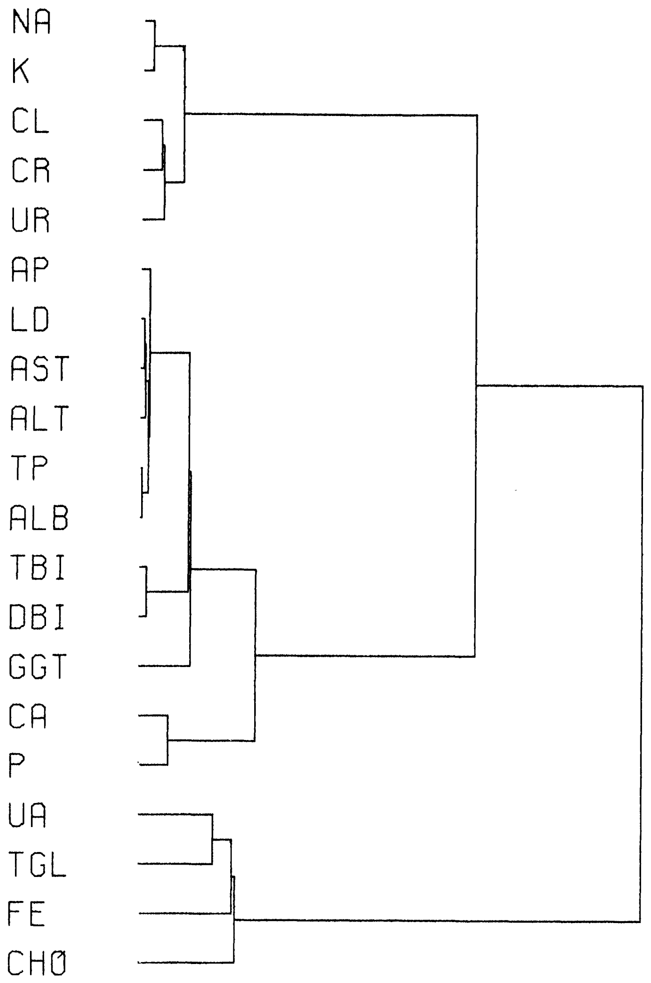

*Figure 7(a). Clusters in test requests of the hepatology out-patient department.*

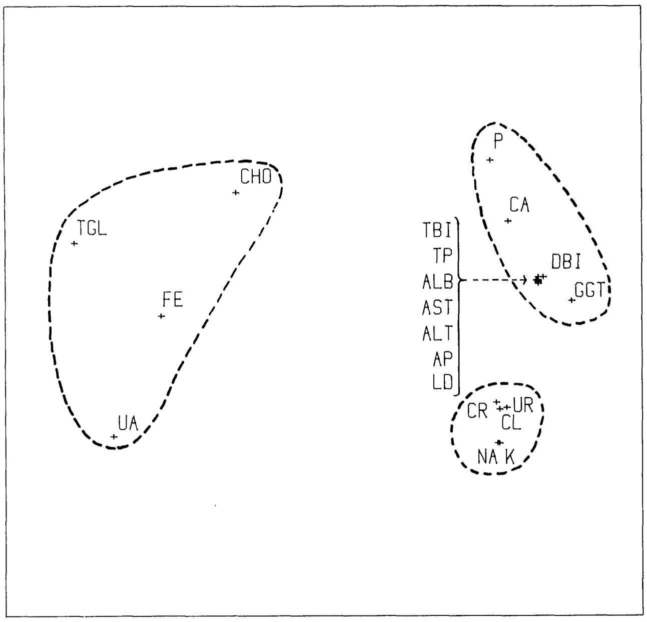

*Figure 7(b). Multidimensional scaling of test requests of hepatology out-patient department.*

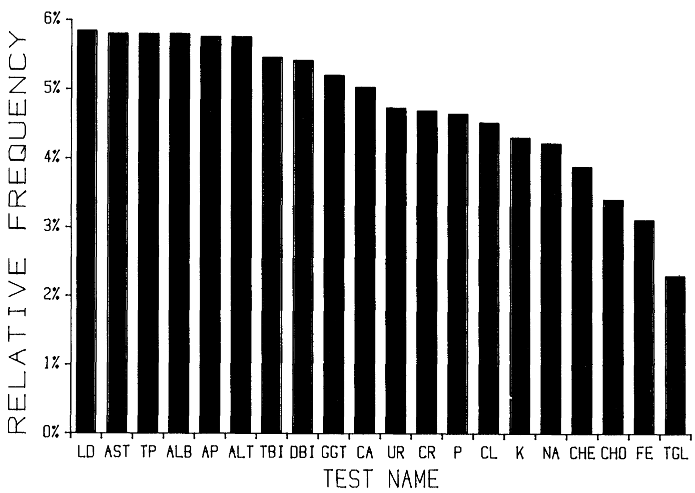

*Figure 7(c). Frequency histogram of test requests of the hepatology out-patient department.*

The requests for hepatology out-patients (figure 7) also show a main clustering of a set of rarely ordered tests, and another of the more frequently requested ones. Again, the clustering is visible in the frequency diagram, but less clearly than in the previous case. Uric acid, triglycerides, iron and cholesterol are similar in receiving relatively little attention from the hepatologists. The kidney function tests form the strongest cluster. LD, AST, total protein, albumen, AP and ALT are not only the tests most frequently ordered by heptatologists, they are also generally requested together, which usage is founded in liver pathology, forming a strong sub-cluster. The MDS plot confirms the dendrogram results. The differences with the cardiology structure are evident.

The main impression of the nephrology test requests (figure 8) again is a cluster containing less frequently ordered tests, and another embracing the more common ones. Subclusters are also reflected in the steps of the frequency diagrams to a large extent. The kidney function cluster is present again. The elements of this cluster are the most frequently ordered tests for this department, as expected. The clustering within the group of less frequently requested tests is only vague, suggesting large variation in individual cases. The MDS plot (figure 8[*b*]) shows a very tight group including the kidney function tests and CA, P, TP and ALB. The cluster to which AP belongs is far less clear from this plot than suggested in the dendrogram. All other tests seem to be selected individually only if needed.

### Conclusions

Various sections of the Department for Internal Medicine order laboratory tests with different frequency.

In the first steps of clustering, when the tightest clusters are formed, the differences between various department sections in requesting laboratory tests are very small. Especially tests for total and direct bilirubin, ALT and AST, and sodium, potassium, urea and creatinine are very often treated as inseparable in the request.

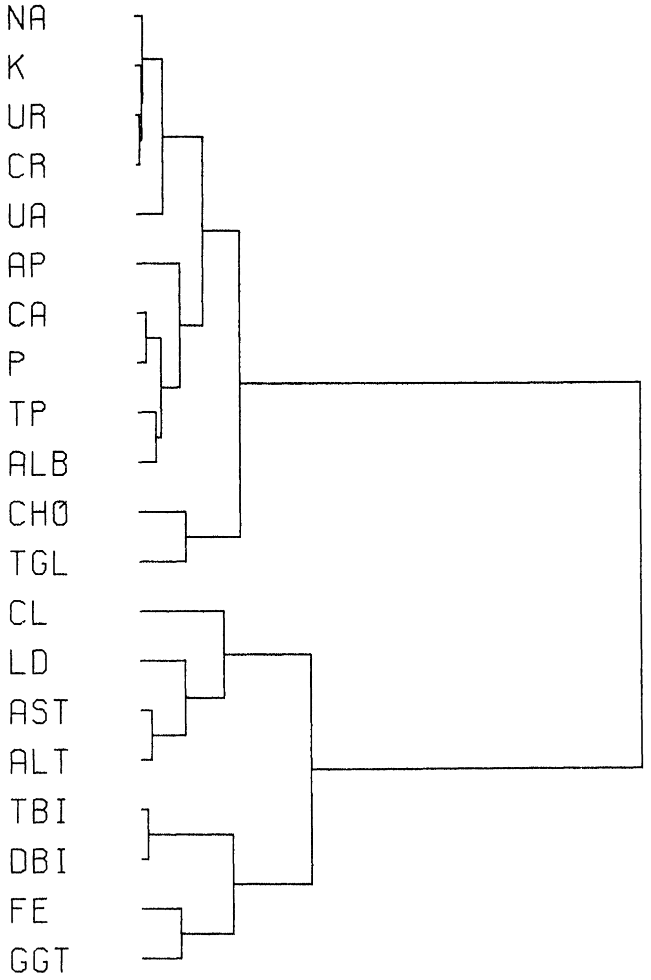

*Figure 8(a). Clusters in test requests of the nephrology out-patient department.*

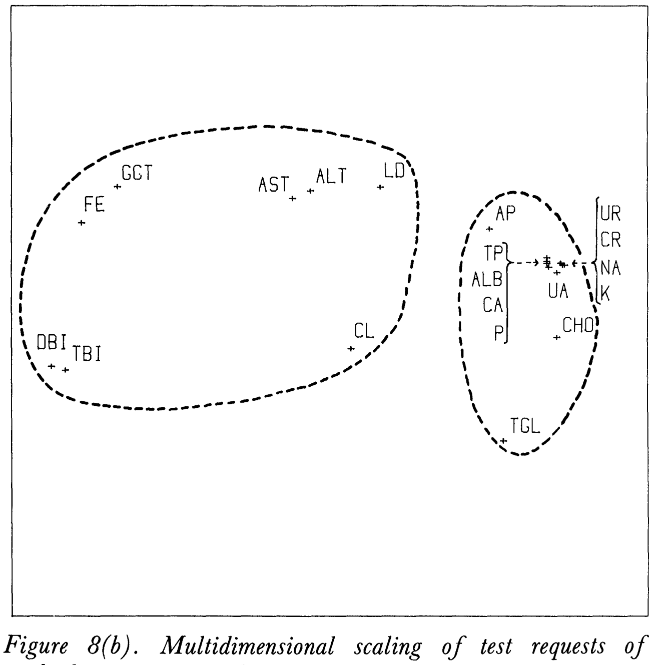

*Figure 8(b). Multidimensional scaling of test requests of nephrology out-patient department.*

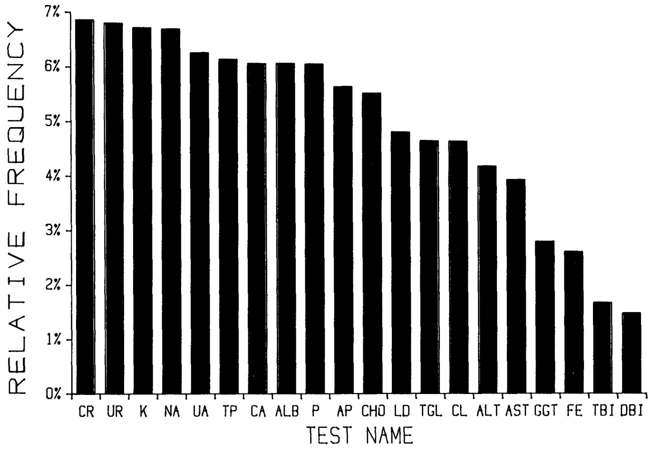

*Figure 8(c). Frequency histogram of test requests of the nephrology out-patient department.*

Large differences are found in the last agglomerations of the clustering process: the global clusters turn out to be very much in accordance with frequency of requesting. If the tests are sorted in order of decreasing frequency, the frequency histogram shows more or less clear levels for subgroups of tests. The steps suggest the regular use of schemes in requesting tests. That these steps are not seen in the merged out-patient data may either indicate that schemes are used less often, or be caused by the different schemes in use in the various out-patient departments. Systematization of test requests seems to be much more uniform throughout the in-patient departments considering the tight clusters seen there. It can be concluded that test results in sets of records of patients from various department sections are not missing at random. Missing-data handling techniques based on the assumption of random occurrence of data gaps are therefore not appropriate in studying such data sets.

If dendrograms are used to represent clusters, a wrong impression may be given of the mutual similarity between members of different clusters. This impression can be corrected by looking at MDS-plots. However, MDS-plots include some distortion of the distances between the points. This poses restrictions to the conclusions that can be drawn from these displays.

In this study several techniques, ranging from simple frequency counts to more complex MDS plots and cluster analyses, were applied to describe patterns in test requests. Although many results obtained by these analyses may seem obvious, it is useful to establish objectively what was only suspected on a subjective basis. A profitable use may be in observing changes in the request patterns, which are now more accessible for control and correction where appropriate. The method followed in this study is not adequate to study changed *needs* for standard test-packages. It seems likely that the available package of routine tests conserves itself. Only large discrepancies between offered and wanted packages will show by the occurrence of tight clusters consisting of mixtures of parts of several offered test packages.

The use of techniques like multi-dimensional scaling and cluster analysis is restricted by the difficulty of judging the statistical significance of the observed phenomena.

For this purpose in some cases appropriate techniques are offered by classical statistics. Display techniques, like the ones described in this paper, can suggest the existence of phenomena before they are strong enough to be concluded significant. Early detection may stimulate further and more specific research to establish the significance of these phenomena.

### References

1. Varmuza, K., *Pattern Recognition in Chemistry* (Lectures notes in chemistry 21, Springer, Berlin, 1980), 26.
2. Everitt, B., *Cluster Analysis* (Heinemann Educational Books, London, 1974).
3. Wishart, D. *CLUSTAN 2.1 User Manual* (Program Library Unit, Edinburgh University, Edinburgh, 1982).
4. Coomans, D and Massart, D. L., *Analytica Chimica Acta*, **133** (1981), 225.
5. Wilmink, F. M. and Uytterschaut, H. T., in *Multivariate Statistical Methods in Physical Anthropology*. Ed. Van Vark, G. N. and Howells, W. W. (D. Reidel, 1984), p. 135.
6. Massart, D. L., Plastria, F., Kaufman, L. and Coomans, D., in *Symposium Anvendt Statistik*, Ed. Höskuldsson, A., Conradsen, K., Jensen, B. S. and Esbensen, K. (NEUCC-RECAU-RECKU, Copenhagen, 1981).
7. Ward, J. H., *Journal of the American Statistical Association*, **58** (1963), 236.
8. Coxon, A. P. M., *The User's Guide to Multidimensional Scaling* (Heinemann Educational Books, London, 1982).
9. Kruskal, J. B., *Psychometrika*, **29** (1964) 1.
10. Leguit, F. A., in *Man and Information Technology:* Towards Friendlier Systems, Ed. Van Apeldoorn, J. H. F. (STT Publications vol. 38, Delft University Press, Delft, 1983), 35.
11. Wilkinson, L., *SYSTAT: the System for Statistics* (SYSTAT Inc., Evanston IL, 1986).

### EUROANALYSIS VII

**Vienna, Austria**

**August 26–31 1990**

Euroanalysis VII will be held at the Technical University, Vienna, Austria in August 1990. The conference will emphasize the role of analytical chemistry for problem solving in major areas of sciences as well as methodological developments. Special sessions and workshops will be held on sampling and sample preparation, computer based analytical chemistry, quality assurance in analytical chemistry and new trends in teaching analytical chemistry.

The final date for receipt of abstracts in 31 January 1990.

*For further information, contact: Euroanalysis VII, Prof. Dr M. Grasserbauer, c/o Interconvention, Austria Center Vienna, A-1450 Vienna, Austria. Tel.: +43 222 2369 2647. Telex: 11 18 03. Telefax: +43 222 2369 648.*

### NOTES FOR AUTHORS

*Journal of Automatic Chemistry* covers all aspects of automation and mechanization in analytical, clinical and industrial environments. The *Journal* publishes original research papers; short communications on innovations, techniques and instrumentation, or current research in progress; reports on recent commercial developments; and meeting reports, book reviews and information on forthcoming events. All research papers are refereed.

### Manuscripts

Two copies of articles should be submitted. All articles should be typed in double spacing with ample margins, on one side of the paper only. The following items should be sent: (1) a title-page including a brief and informative title, avoiding the word 'new' and its synonyms; a full list of authors with their affiliations and full addresses; (2) an abstract of about 250 words; (3) the main text; (4) appendices (if any); (5) references; (6) tables, each table on a separate sheet and accompanied by a caption; (7) illustrations (diagrams, drawings and photographs) numbered in a single sequence from 1 upwards and with the author's name on the back of every illustration; captions to illustrations should be typed on a separate sheet. Papers are accepted for publication on condition that they have been submitted only to this *Journal.*

### References

References should be indicated in the text by numbers following the author's name, i.e. Skeggs [6]. In the reference section they should be arranged thus:

to a journal

Manks, D. P., *Journal of Automatic Chemistry*, **3** (1981), 119.

to a book

Malmstadt, H. V., in *Topics in Automatic Chemistry*, Ed. Stockwell, P. B. and Foreman, J. K. (Horwood, Chichester, 1978), p. 68.

### Illustrations

Original copies of diagrams and drawings should be supplied, and should be drawn to be suitable for reduction to the page or column width of the *Journal*, i.e. to 85 mm or 179 mm, with special attention to lettering size. Photographs may be sent as glossy prints or as negatives.

### Proofs and offprints

The principal or corresponding author will be sent proofs for checking and will receive 50 offprints free of charge. Additional offprints may be ordered on a form which accompanies the proofs.

*Manuscripts should be sent to Dr P. B. Stockwell, P.S. Analytical Ltd, Arthur House, Unit B4, Chaucer Business Park, Watery Lane, Kemsing, Sevenoaks, Kent TN15 6QY, UK.*

